# Ferroptosis: An Emerging Target for Bladder Cancer Therapy

**DOI:** 10.3390/cimb45100517

**Published:** 2023-10-10

**Authors:** Zhengda Shan, Wenbin Tang, Zhiyuan Shi, Tao Shan

**Affiliations:** 1 School of Medicine, Sun Yat-sen University, Shenzhen 518107, China; shanzhengda_jerry@126.com; 2 School of Medicine, Xiamen University, Xiamen 361102, China; tangwenbintwb@163.com; 3 School of Basic Medicine, Qingdao University, Qingdao 266071, China

**Keywords:** bladder cancer, ferroptosis, mechanism, gene, drug

## Abstract

Bladder cancer (BC), as one of the main urological cancers in the world, possesses the abilities of multiple-drug resistance and metastasis. However, there remains a significant gap in the understanding and advancement of prognosis and therapeutic strategies for BC. Ferroptosis, a novel type of iron-dependent regulated cell death, depends on lipid peroxidation, which has been proven to have a strong correlation with the development and treatment of BC. Its mechanism mainly includes three pathways, namely, lipid peroxidation, the antioxidant system, and the iron overload pathway. In this review, we reviewed the mechanism of ferroptosis, along with the related therapeutic targets and drugs for BC, as it might become a new anticancer treatment in the future.

## 1. Introduction

Bladder cancer (BC) is one of the major malignant tumors in the urinary system. There were approximately 573,000 new cases and 213,000 deaths in 2020, with a male-to-female ratio of approximately 4:1 [1]. Smoking has been identified as a primary risk factor, while other factors such as schistosoma japonicum infection and arsenic exposure are also associated with an increased likelihood of developing BC [2]. The mortality rate in more developed countries has declined due to the improvement of treatment methods [3]. According to the depth of invasion, BC can be divided into two types: non-muscular invasive BC (NMIBC) and muscular invasive BC (MIBC). Without appropriate intervention, the risk of death of BC patients is relatively high. In Europe, the standard relative five-year survival rate of BC patients is lower than 60%, and the five-year survival rate is reduced to 5.5% after metastasis [1,4]. In a word, early diagnosis and treatment are crucial for improving outcomes in BC patients.

Ferroptosis, an iron-dependent regulated cell death, is a modifiable and complex programmed cell death that was coined in 2012 [5]. Distinct from other forms of cell death such as apoptosis, necrosis, and autophagy, ferroptosis depends on an imbalance of reactive oxygen species (ROS) production and degradation to induce phospholipid peroxidation and its product’s accumulation. Consequently, characteristic changes occur in cells, including cell membrane rupture and various changes in mitochondria such as atrophy, cristae deletion, and increased membrane density [6,7]. Ferroptosis can be triggered through endogenous or exogenous pathways. The exogenous pathway is initiated by the inhibition of cell membrane transporters such as the cystine/glutamic acid transporter (also known as system ${X_{c}}^{-}$) or the activation of the iron transporter, serotonin transporter, and ferritin [8]. Activation of the intrinsic pathway relies on blocking intracellular antioxidant enzymes (e.g., glutathione peroxidase 4 (GPX4)) [9,10]. Given the high uptake of iron by cancer cells, ferroptosis might play a key role in tumor progress [11]. At present, several studies have reported that ferroptosis and its regulatory genes can determine the fate of BC cells [12,13]. In this review, we reviewed the mechanism of ferroptosis and its effect on BC. We deem that targeting ferroptosis might be a novel therapy for BC in the future.

## 2. Mechanism of Ferroptosis

**Figure 1:**
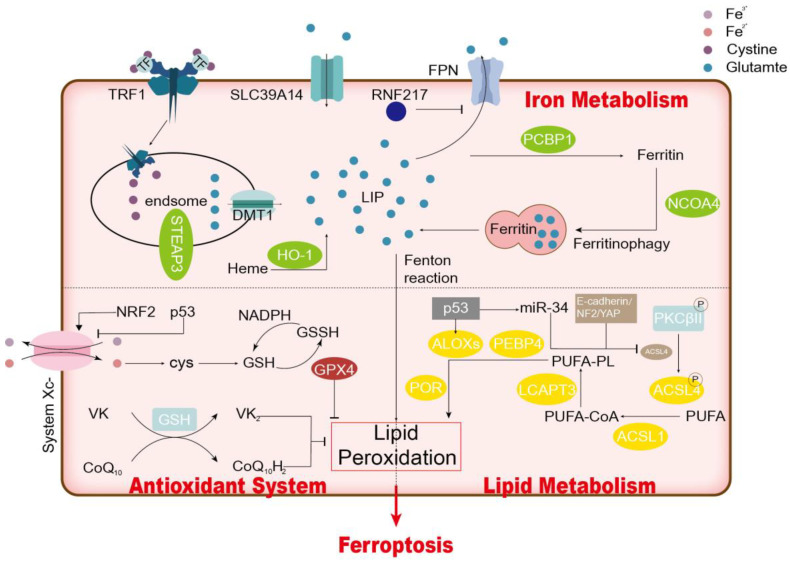
Mechanism of Ferroptosis.

The main molecular mechanisms of cellular ferroptosis are lipid oxidation, antioxidant imbalance, and iron overload (Figure 1). Lipid peroxidation might be the core driver of ferroptosis.

### 2.1. Lipid Peroxidation

A trait of ferroptosis is uncontrolled lipid peroxidation, which leads to the destruction of the lipid bilayer and the impairment of cell membrane function [14]. What has gained consensus is that the degree of cellular sensitivity to ferroptosis is critically dependent on the degree of unsaturation of the lipid bilayer. The initiation of lipid peroxidation requires the removal of bisallylic hydrogen atoms (located between two carbon–carbon double bonds) from the polyunsaturated fatty acyl moieties of phospholipids (PUFA-PLs), particularly arachidonic acid and adrenic acid bound to the lipid bilayer. This process leads to the formation of a phospholipid radical (PL•) centered on carbon, which subsequently undergoes a reaction with molecular oxygen to generate a phospholipid peroxy radical (PLOO•). PLOO• further removes hydrogen from another PUFA to form PLOOH [15]. If PLOOH fails to be converted into the corresponding alcohol (PLOH) by GPX4, PLOH and a lipid radical (especially an alkoxy phospholipid radical (PLO•)) react with PUFA-PLs. The production of PLOH is propagated by further removing hydrogen atoms, reacting with molecular oxygen, and forming PLOH. The end result is a large number of secondary products forming, including decomposition products of lipid peroxide and oxidized and modified proteins. This chain reaction might eventually lead to the destruction of membrane integrity and the rupture of organelles and/or cell membranes. The inactivation of acyl-CoA synthase long-chain family member 4 (ACSL4) and lysophosphatidylcholine acyltransferase 3 (LPCAT3) confers resistance to different GPX4 inhibitors in these cells [5]. They are involved in the activation and integration of PUFAs, such as the transformation of arachidonic acid (AA) into membrane-located lipids [16]. ACSL4 catalyzes the conjugation of free arachidonic acid or adrenergic acid to CoA to generate the derivatives AA-CoA or AdA-CoA, separately, whose esterification is subsequently stimulated to membrane phosphatidylethanolamines to form AA-PE or AdA-PE by LPCAT3. These results indicate that unsaturated fatty acids need to exist in their membrane-bound environment to show lethality after peroxidation. In the context of lipid peroxidation, the involvement of various lipoxygenases in the generation of hydroperoxides like AA-PE-OOH or AdA-PE-OOH plays a crucial role in triggering ferroptosis [17]. However, the specific impact of this process depends on the particular circumstances. For instance, the phosphorylation of Acetyl-CoA carboxylase (ACAC) by lipoxygenases such as ALOX5, ALOX3, ALOX15, and ALOX15B is believed to hinder ferroptosis by limiting the production of PUFA, thereby exerting an inhibitory effect.

### 2.2. Antioxidant Mechanism

GPX4, also known as phosphatide hydrogen peroxide glutathione peroxidase, is a selenoprotein that serves as the main enzyme that catalyzes the reduction of PLOOHs in human cells [9,18]. Its expression and activity rely on the presence of GSG and selenium. Most cancer cells mainly mediate cystine uptake through system ${X_{c}}^{-}$. Solute carrier family 7 member A11 (SLC7A11) is the main functional subunit of system ${X_{c}}^{-}$, which is responsible for transporting cystine into cells. The expression and activity of SLC7A11 is further controlled by dual intracellular regulation, resulting in a mechanism of fine-tuning of GSH levels in ferroptosis. NFE2L1 upregulates its expression, while tumor suppressor genes, such as TP53, BAP1, and BECN1, inhibit its activity. Cystine could be reduced to cysteine in cells [19,20,21,22,23]. Cysteine and glutamic acid are both utilized for the biosynthesis of reduced glutathione (GSH), a thiol-containing tripeptide derived from glycine, glutamate, and cysteine. Through the catalytic selenocysteine residue of GPX4 and two electrons provided by GSH, GPX4 could reduce phospholipids and cholesterol hydroperoxide to the corresponding alcohols [24]. Inhibition of system ${X_{c}}^{-}$ or GPX4 with drugs like erastin or RSL3 could induce ferroptosis [15]. Also, the antioxidant system would be suppressed by the genetic depletion of SLC7A11 or GPX4, which further causes lipid peroxidation [25,26]. In the Gpx4 knockout mouse model, erastin could prevent cystine uptake, leading to the accumulation of PLOOH, irreparable rapid damage to the membrane, and ultimately ferroptosis. Thus, the antioxidant system formed by the SLC7A11-GSH-GPX4 axis constitutes the main cellular system to resist ferroptosis. Other alternative pathways, such as the AIFM2–CoQ10, GCH1–BH4, and ESCRT-III membrane repair system, also exhibit context-dependent functions in safeguarding against oxidative damage in the process of ferroptosis [27,28,29,30].

### 2.3. Iron in Ferroptosis

Compared to normal cells, the proliferation of cancer cells has a strong demand for the trace element iron. The non-enzymatic iron-dependent Fenton chain reaction might be the key to ferroptosis: when GPX4 is inhibited, PLOOHs can persist for an extended period, and the Fenton reaction can be initiated to rapidly amplify PLOOHs, which is the sign of ferroptosis [15]. Ferrous or ferric ions can both react with PLOOHs to form PLO• and PLOO•, which trigger a destructive chain reaction of peroxidation. Furthermore, iron is essential for the catalysis activity of lox and POR. Iron is also involved in numerous redox metabolic processes that contribute to the production of ROS within cells. Various cellular processes modulate the sensitivity of cells to iron metamorphosis by regulating the soluble iron content in the cells. Enhanced iron export and import have been shown to inhibit or induce ferroptosis. For example, prominin2, a pentaspanin protein implicated in the regulation of lipid dynamics, inhibits ferroptosis by facilitating the formation of multivesicular bodies (MVBs) and exosomes of iron-containing proteins, thereby transporting iron efflux from the cell [31]. Instead, iron uptake mediated by serum transferrin or lactotransferrin promotes ferroptosis through TFRC [32,33], while iron output mediated by Ferroportin (Fpn) inhibits ferroptosis [34,35,36,37,38]. There have also been experiments showing that in the absence of transferrin expression, liver cells compensate by upregulating the expression of the metal transporter protein SLC39A14 to excessively take up iron, thereby driving ferroptosis [39]. Autophagic degradation of ferritin (an iron storage protein) could enhance ferroptosis by increasing the intercellular iron level [7,33], while extracellular-mediated ferritin export inhibits ferroptosis [31]. Iron overload activates lipid peroxidation by producing ROS in an iron-dependent Fenton reaction and activating iron-containing enzymes (e.g., lipoxygenase) [40]. Thus, iron-chelating agents and antioxidants may prevent ferroptosis.

### 2.4. Other Regulators of Ferroptosis

#### 2.4.1. NRF2

Nuclear factor erythroid 2-related factor 2 (NRF2) serves as the primary regulator of antioxidant responses, as it is upregulated in response to the accumulation of ROS. This activation leads to transcriptional responses that inhibit ferroptosis [7]. The transcription of numerous genes related to the GPX4-GSH-mediated defense against ferroptosis, such as SLC7A11, is regulated by NRF2, thereby promoting the evasion of ferroptosis by cancer cells. As a result, NRF2 signaling is upregulated in various human cancer types [23,41,42]. The specific mechanisms through which NRF2 provides protection vary depending on the cell and tissue context. For example, NRF2 counteracts ferroptosis by activating microsomal glutathione S-transferase 1 (MGST1), which inhibits ALOX5 in pancreatic cancer cells [43]. A knockdown of NRF2 augments the susceptibility of head and neck cells to artesunate-induced ferroptosis. In liver cancer cells, NRF2 promotes resistance to ferroptosis by regulating ferritin levels [7]. BC cells with increased expression of NRF lead to an upregulation of GCLM, which helps in the synthesis of glutathione to counteract ferroptosis [42,44].

#### 2.4.2. p53

p53 regulates key functions of tumor genes including the induction of cell cycle arrest, cellular senescence, or apoptosis. It is involved in the regulation of carcinogenesis by modulating ferroptosis in cancer cells [45,46]. p53 may have pro- or anti-ferroptosis effects on oxidative stress. p53 might act as a rheostat, preventing ferroptosis under basal or low ROS stress and promoting ferroptosis under high oxidative stress [47,48]. Under cellular stress conditions, p53 inhibited cystine uptake by transcriptionally suppressing SLC7A11. The regulation of the expression of SLC7A11 required the acetylation of the DNA-binding domain of p53. p53 could also regulate ferroptosis via its metabolic target gene spermidine/spermidine N1-acetyltransferase (SAT1), which encodes a protein involved in the polyamine metabolic pathway and is widely downregulated in human tumors. Knocking out SAT1 could significantly eliminate p53-mediated ferroptosis, while the increased expression of SAT1 made cells sensitive to ferroptosis under ROS stress [49].

#### 2.4.3. FSP1

Ferroptosis suppressor protein 1 (FSP1) was initially referred to as p53-responsive gene 3 (PRG3) due to its association with the p53 pathway [50]. As an obvious ferroptosis inhibitor, FSP1 is a target of the transcription factor NRF2, which is localized on the plasma membrane [51]. It mainly helps cells resist ferroptosis by strengthening the antioxidant system. FSP1 could reduce ubiquinone (or its partially oxidized product semi-hydroquinone) to generate panthenol (which could directly reduce lipid free radicals to stop lipid autooxidation) or indirectly inhibit lipid peroxidation and ferroptosis by regenerating oxidized α-tocopherol free radicals (vitamin E)— powerful natural antioxidants [27,28]. Acting as an NAD(P)h-dependent oxidoreductase, FSP1 is capable of reducing ubiquinone (also known as coenzyme Q or CoQ) to ubiquinol (CoQH2) [27,28,52,53]. In addition to its well-established role in mitochondrial electron transport, CoQH2 can effectively scavenge lipid peroxyl radicals, thus suppressing lipid peroxidation and ferroptosis. This protective effect of ubiquitin revealed why some cells and tissues, such as liver cells with high metabolic activity, contain a substantial amount of exomitochondrial ubiquinone, which is inconsistent with its typical role in the mitochondrial electron transport chain [54].

#### 2.4.4. E-Cadherin-NF2-Hippo-Yap Signaling Pathway in Ferroptosis

This pathway holds important biological significance, including its role in regulating cell growth and the size of organs [55,56]. E-cadherin-mediated cell–cell contacts could mediate the cell density effect on ferroptosis in epithelial cells. This effect could stimulate Hippo signaling via NF2 and suppress the activity of YAP, whose downstream molecule could affect ferroptosis in cancer cells, such as ACSL4, and transferrin receptor 1. That pathway could exert a substantial influence on ferroptosis susceptibility. According to data from The Cancer Genome Atlas (TCGA), mutation of the tumor suppressor E-cadherin, leading to a loss of function, is a common occurrence in breast lobular invasive carcinoma (approximately 65%) and diffusive gastric adenocarcinoma (about 25%) as well as other types of cancer. Similarly, loss of function mutation of NF2 is observed in more than 30% of mesothelioma cases and all cases of a group of benign diseases known as NF2 diseases. Significantly, malignant mutations of these genes typically contribute to metastasis, confer resistance to apoptosis and ferroptosis in cancer cells, and enhance their resistance to conventional cancer treatment [57,58]. Therefore, understanding the interaction between the signaling pathway and ferroptosis metabolism in cancer may pave the way for novel approaches to cancer treatment [59,60].

#### 2.4.5. AMPK Signaling and Ferroptosis

AMPK was primarily implicated in the regulation of energy to counteract lipid peroxidation, a crucial process in ferroptosis. Intuitively, energy and metabolic stress should result in the depletion of energy and the cascading failure of the systems needed to maintain homeostasis, such as energy-dependent ion gradients across cell membranes, ultimately resulting in cell death [61]. Additionally, metabolically stressing cells with glucose starvation increased ROS production, suggesting that glucose starvation promoted ferroptosis [62]. However, after glucose starvation, energy-sensing kinase and AMPK activation may actually inhibit the occurrence of ferroptosis [17]. Consequently, in the face of glucose deficiency, cells opt to activate AMPK, opening an energy stress protection scheme against ferroptosis that involves the impaired biosynthesis of PUFAs, which are essential for lipid-peroxidation-driven ferroptosis [40,63]. The effect of AMPK depends on the phosphorylation of different downstream substrates. The phosphorylation of beclin 1 by AMPK enhances ferroptosis by suppressing the synthesis of reduced GSH [22]. Conversely, AMPK-mediated phosphorylation of ACAC has been proposed to impede ferroptosis by restricting the production of PUFAs [17].

## 3. The Role of Ferroptosis in BC

### 3.1. RNAs

It has been reported in many studies that RNA containing lncRNA and miRNA participates in various biological activities such as metabolism, infection, and immune responses. Several studies have shown a significant association between RNA and the process of ferroptosis in different types of malignant tumors [64,65,66,67]. Some RNAs promote the occurrence of ferroptosis, while others inhibit ferroptosis (Table S1). For example, lncRNA RP11-89 could restrain ferroptosis by “sponging” miR-129-5p and promoting the expression of PROM2. Iron export was stimulated by the miR-129-5p/PROM2 axis. In this way, BC cells escape ferroptosis [13]. One study showed that upregulated circST6GALNAC6 could lead to the ferroptosis of BC cells via the small heat shock protein 1 (HSPB1)/p38 MAPK pathway [68]. This circRNA activated the p38 MAPK signaling pathway and blocked the phosphorylation of the Ser-15 site of HSPB1, which was the phosphorylation site in ferroptosis antagonism, to show a stimulated effect on cell ferroptosis. The miRNA could also influence ferroptosis through exosomes. For instance, miR-217 transported by BC-tissue-derived exosomes would prevent ferroptosis of cells, leading to carcinogenesis and enhanced cancer cell proliferation and migration [69]. Additionally, Fin56 is a type 3 ferroptosis inducer that could accelerate ferroptosis in BC cells by promoting the degradation of GPX4 protein [12]. Moreover, Fin56 and Torin 2 (a potent mTOR inhibitor) could synergistically reduce the viability of cancer cells.

Despite the existence of available treatments, there is a high incidence of MIBC recurrence, progression, and mortality [70]. For precise treatment, many scholars have established related models to predict the prognosis of patients based on ferroptosis-related RNA in BC [71,72,73,74]. These prognostic models might become the key to individualized treatment. We could use the model constructed using lncRNAs to predict the risk of BC. For example, an 11-lncRNA signature (AL031775.1, AC018653.3, AC011468.1, AL583785.1, AC021321.1, AP003352.1, ‘ETV7-AS1’, U47924.1, AC010326.3, LINC02762, and 18S) was used to divide patients into high- and low-risk groups [71]. The frequency of ferroptosis and the expression of m6A methylation-related genes were different between the two risk groups, which could predict the prognosis of BC patients.

### 3.2. Genes

Numerous studies have investigated the correlation between ferroptosis-related genes and the occurrence, development, and prognosis of BC. Certain genes have been identified as inhibitors of ferroptosis in BC cells and are known to promote cancer progression. For instance, GCLM, which was highly expressed in BC cells, could significantly enhance the colony formation ability and played an important role in immune infiltration [75]. Previously, Harris et al. demonstrated the pivotal role of the GCLM-mediated synthesis of the antioxidant glutathione (GSH) in tumor development [76]. Inoue et al. reported that targeting GCLM could enhance the process of overcoming cisplatin resistance in non-small-cell lung cancer, suggesting its potential as a therapeutic target [77]. Additionally, it could repress ferroptosis in tumor cells by restraining the accelerative influence of Anti-PD-L1 antibodies on NK T cells. WTAP, the N6-methyladenosine (m6A) methyltransferase (‘writer’), could make BC cells more viable and act as an erastin-induced ferroptosis inhibitor [78,79]. It could display a depressant effect on ferroptosis by influencing the expression and stability of the endogenous antioxidant factor NRF2 via m6A. The expression of Epithelial Membrane Protein 1 (EMP1) is restrained in BC cells, which would make cells more viable and inhibit ferroptosis [80,81]. A lack of EMP1 could upregulate and activate PPARG, resulting in the stimulation of the expression of pFAK (Y397) and SLC7A11. Thus, BC cells could be more likely to survive and migrate. The association between the abnormal expression of heat shock protein family A (HSP70) member 5 (HSPA5) and the progression and prognosis of cancer in multiple tumors has been a focus in cancer research [82,83,84,85]. A recent study showed that ferroptosis in BC was inhibited by HSPA5 via the P53/SLC7A11/GPX4 pathway. In addition, it could also promote the proliferation, migration, and invasion of BC cells by regulating the VEGFA/VEGFR2 signaling pathway. The expression of Poly C Binding Protein 1 (PCBP1), a ferroptosis-related regulator that serves as an iron chaperone, increased significantly in two BC cell lines (T24 and UMUC3), which could inhibit erastin-mediated ferroptosis [86,87,88,89]. It could influence ferroptosis via the serine β-lactamase-like protein (LACTB)/ phosphatidylserine decarboxylase (PISD) axis [90]. A knockdown of PCBP1 could strengthen the anticancer effect of sulfasalazine, leading to an increase in LACTB and a decrease in PISD.

On the contrary, some genes could induce ferroptosis by regulating the protein expression or drug sensitivity of cancer cells and finally reduce the survival of BC cells. The staphylococcal nuclease and tudor domain containing 1(SND1)-GPX4 axis could affect the drug susceptibility of BC cells. SND1 is considered a transcription factor that plays a role in various post-transcriptional regulatory activities [91]. Silencing SND1 and GPX4 might make cells sensitive to a chemotherapeutic like cisplatin, which would enhance ferroptosis in BC cells [92]. Excessive expression of GPX4 could counteract those effects. PhosphoGlycerol Dehydrogenase (PHGDH), which was over expressed, could inhibit ferroptosis in BC cells [93]. PHGDH stabilized PCBP2, an RNA-binding protein that could upregulate the expression of SLC7A11. PHGDH inhibitors such as NCT-502 could enhance ferroptosis to suppress the malignant progression of cancer via an interaction between PHGDH and PCBP2 [94]. Among various lipoxygenases, ALOX15B was significantly downregulated in BC tissues. It could regulate ferroptosis under the influence of p53 [95]. p53 could inhibit the SLC7A11-activated lipoxygenase activity of ALOX15B to induce ferroptosis in BC cells. Fibronectin leucine rich transmembrane protein 2 (FLRT2) is related to the regulation of the progression of various tumors, including BC, as a tumor suppressor gene [96,97]. The downregulation of FLRT2 could stimulate BC cell growth, migration, and invasion [96]. Furthermore, the study showed that FLRT2 could upregulate the expression of ACSL4, increase lipid peroxidation, and eventually lead to ferroptosis [63]. Overexpression of glutathione S-transferase zeta 1 (GSTZ1), whose expression was repressed in BC cells, could decrease GPX4 and GSH and upregulate the contents of iron, MDA, ROS, and transferrin [98]. In addition, it stimulated HMGB1/GPX4 signaling [99,100]. Via the above pathway, GSTZ1 overexpression induced ferroptosis to inhibit proliferation in BC cells.

The tumor microenvironment (TME) has consistently been a focal point in BC research, and it plays an important role in BC progress and metastasis. The epithelial–mesenchymal transition (EMT) is an indispensable mechanism [101,102]. In addition, calumenin (CALU) is regarded as a symbolic gene of the EMT process that impacts cancer metastasis in various types of tumors [103,104,105]. Research by Du, Y. et al. revealed positive correlations between CALU and cancer-associated fibroblasts (CAFs), CD8+ T cells, and macrophages, and it was relevant to numerous immune-checkpoint-related genes [106]. Meanwhile, the result showed that CALU might regulate ferroptosis by affecting ferroptotic gene mutations including TP53.

Similarly, prognostic models based on ferroptotic genes have also been developed [107,108,109,110]. Ferroptosis might have different impacts on different types of BC. In MIBC and NMIBC, different signatures were constructed separately. A seven-gene signature (GCLM, CRYAB, SLC3A2, TFRC, SQLE, G6PD, and ACSF2) could provide a prediction about OS in MIBC patients. In NMIBC, a six-gene signature (FANCD2, PTGS2, AIFM2, ACSL3, FADS2, and ABCC1) could predict recurrence-free survival (RFS). Zhang and his team also revealed differences in patient characteristics between groups at risk for genes associated with ptosis and between MIBC and NMIBC. These results suggest that ferroptosis may play distinct roles in the development of MIBC and NMIBC [107].

### 3.3. Drugs

In light of the current research on the pathways and targets associated with ferroptosis, such as GPX4, scholars have conducted extensive studies on drugs to promote the ferroptosis of BC cells and inhibit their progress. Baicalin is an effective component of Scutellaria baicalensis Georgi against cancer [111]. Not only could it promote apoptosis and cell death, but it was also a significant ferroptosis inducer in BC cells. Baicalin would upregulate FTH1, which was a crucial promoter in the process of ferroptosis, and induce the accumulation of ROS and iron in bladder cells to achieve an anticancer effect. Erianin exacted from Dendrobium chrysotoxum Lindl could induce cell death and cell cycle arrest in BC cells [112]. Ferroptosis via NFR2 might be a vitally important part of cell death induced by Erianin, through which Erianin played an anticancer role and stopped tumors from growing in vivo. Bupivacaine, a common anesthetic, could also suppress the development of BC by guiding cancer cells to apoptosis and ferroptosis [113]. Bupivacaine deadened the phosphorylation of PI3K, Akt, and mTOR [114]. In other words, it induced ferroptosis in BC cells by downregulating the expression of the PI3K/AKT signaling pathway. Abietic acid (AA), a kind of abietane diterpene with the ability to hinder the progress of BC, selectively targets cancer cells without affecting normal cells [115,116]. The expression of GPX4 was downregulated and HO-1 was upregulated under the influence of AA [117]. As a result, an increasing number of BC cells would deactivate because of ferroptosis. Some scholars have synthesized a series of quinazolinyl urea derivatives based on the structural modification of the molecular targeted drug sorafenib [118,119,120]. Among them, 7j could induce intracellular ROS production and lead to ferroptosis by binding to the active site of the corresponding receptor (GPX4) [121]. Another type 3 ferroptosis inducer, Fin56, may bring out ferroptosis by influencing GPX4 degradation [122]. But how Fin56 boosts this pathway was not clear before. Yadong Sun et al. confirmed that Fin56 induced both ferroptosis and autophagy in BC cells [12]. And what is more important is that the autophagic machinery is a vitally significant part of Fin56-triggered ferroptosis. The combination of Fin56 and Torin 2, a potent mTOR inhibitor, could enhance cytotoxicity to strengthen the curative effect. Hu, C.Y. et al. confirmed the relationship between the anticancer activity of evodiamine (EVO) and ferroptosis [123], as the underlying mechanism was not clear before [124,125]. The GPX4 expression level in BC, which reduces the accumulation of lipid peroxides, was downregulated dose-dependently by EVO treatment. The iron chelator deferoxamine (DFO), an inhibitor of ferroptosis, could lower the lipid peroxide level when used prior to EVO treatment to reduce EVO-induced cell death. In addition, EVO was proven to repress tumor growth and EMT. In brief, it might be a novel drug that can inhibit BC by promoting ferroptosis.

### 3.4. Nanoparticles

It is well recognized that the challenge in the treatment of BC is that it recurs easily, particularly due to the development of resistance to standard intravesical chemotherapy [126]. The treatment scheme using iron oxide nanoparticles (IONPs) to induce ferroptosis, which has been applied in preclinical research, might be a therapy for chemoresistant BC (CRBC) [126,127]. In order to improve the accumulation level of IONPs at a tumor site, Ao Qi and his team presented a three-tier delivery strategy using a mucoadhesive hydrogel platform conveying hyaluronic acid-coated IONPs (IONP-HA) [128]. It includes the following three steps: the hydrogel platform sticks to the surface of the tumor, photothermal therapy, and antibody-mediated endocytosis. By utilizing this mode of administration, the iron content in BC cells is increased by 50 times compared to systematic administration, obviously raising the rate of ferroptosis. Meanwhile, other scholars designed multifunctional nanoparticles to induce ferroptosis and immunoactivation. Yu-Cheng Chin and his group devised a facile one-pot coprecipitation reaction to fabricate cluster-structured nanoparticles (CNPs), which were constructed using Fe_3_O_4_ and iron chlorophyll (Chl/Fe) photosensitizers [129]. Fe_3_O_4_@Chl/Fe CNPs treated BC through both photodynamic therapy (PDT) and chemodynamic therapy (CDT) [5,130]. They are capable of emitting red–NIR fluorescence to induce ROS changes and a Fenton-like reaction, which could lead to ferroptosis. It is worth noting that after PDT-CDT treatment, the tumor microenvironment transformed from immunosuppressive to immunostimulatory. Similarly, to handily fabricate NIR-responsive theranostic materials with quality biocompatibility, Liao, M.Y. chose to use the same model, using Chl/Fe to synthesize Au@Chl/Fe nanorods with prominent stability with the assistance of the J-aggregate as a template [131]. Then, Chl/Fe J-aggregates assembled on a Au surface in large quantities could generate red–NIR fluorescence, enabling the tracing and monitoring of BC lesions. Similar to the aforementioned nanoparticles, this aggregate could also bring out ferroptosis by changing the redox balance and a Fenton-like reaction. Through the method of intravesical instillation of the Au@Chl/Fe-CPBA nanorods, treatment could minimize systemic effects. Another nanoparticle, ZIF-8/PdCuAu/GOx@HA (ZPG@H), is a cascade nanoreactor assembled by the co-encapsulation of PdCuAu nanoparticles and glucose oxidase into zeolitic imidazolate framework-8 (ZIF-8) modified by hyaluronic acid [132]. It could upregulate the ROS level in BC and downregulate mitochondrial depolarization in the tumor microenvironment, which means ZPG@H could induce ferroptosis via starvation therapy and chemodynamic therapy.

## 4. Future and Perspectives

As one of the top ten cancers in the world, the drug resistance and specific refinement of treatment for BC have always been the focus of research in urinary system diseases. As a novel form of cell death, ferroptosis has also been proven to be related to the metastasis, treatment, and prognosis of BC.

Based on the research on genes and RNAs related to the ferroptosis pathway, the constructed model for predicting prognosis and drug resistance has attracted extensive attention. Therefore, it has become possible to accurately predict and guide the treatment of BC after the identification of biomarkers related to ferroptosis in patients. However, currently, there are numerous limitations on ferroptosis in the field of bladder cancer. For instance, related drugs are still in the early stage of clinical research, and there is a lack of clinical data to prove that they can be used in clinical treatment. In addition, the potential toxicity of ferroptosis-inducing drugs on normal organs of the human body, such as the kidney, liver, brain, and heart, is not clear and requires further study.

## 5. Conclusions

It is believed that in the future, after in-depth study on the mechanism and targets of ferroptosis, targeted ferroptosis of BC may become a new anticancer treatment.

## Data Availability

Data sharing not applicable.

## Supplementary Materials

The following supporting information can be downloaded at https://www.mdpi.com/article/10.3390/cimb45100517/s1, Table S1: Inducers/inhibitors of ferroptosis in bladder cancer.
