# Supplementary material for: Ferroptosis: An Emerging Target for Bladder Cancer Therapy
Source: Curr Issues Mol Biol. 2023 Oct 10;45(10):8201–14. doi: 10.3390/cimb45100517 (PMC10605744; doi:10.3390/cimb45100517)
Supplement: Supplementary file 1 [file cimb-45-00517-s001.zip › cimb-2605602-supplementary.pdf]

Table S1. Inducers/Inhibitors of ferroptosis in bladder cancer.

Molecular name	Inducer/Inhibitor of ferroptosis	Target Molecular
**RNA**		
LncRNA RP11-89	Inhibitor	miR-129-5p, PROM2
circST6GALNAC6	Inducer	HSPB1 /p38 MAPK pathway
miR-217	Inhibitor	exosomes
Fin56	Inducer	GPX4
**Gene**		
GCLM	Inhibitor	Anti-PD-L1 antibodies
WTAP	Inhibitor	m6A, NRF2
EMP1	Inhibitor	PPARG, pFAK(Y397), SLC7A11
HSPA5	Inhibitor	P53/SLC7A11/GPX4 pathway
SND1	Inducer	GPX4
PHGDH	Inducer	PCBP2, SLC7A11
ALOX15B	Inducer	p53, ALOX15B
GSTZ1	Inducer	HMGB1/GPX4 signaling
FLRT2	Inducer	ACSL4
**Drug**		
Baicalin	Inducer	FTH1
Erianin	Inducer	NFR2
Bupivacaine	Inducer	PI3K, Akt, mTOR
AA	Inducer	GPX4, HO-1
7j	Inducer	GPX4
EVO	inducer	GPX4
